# Breath-by-breath: unmasking cardio-respiratory conditions through capnography waveforms - the general breathing record study

**DOI:** 10.1080/20018525.2026.2661529

**Published:** 2026-05-25

**Authors:** Daniel M Neville, Rui Hen Lim, Elizabeth Hawke, Leeran Talker, Henry Broomfield, Cihan Dogan, Ahmed B. Selim, Gabriel Lambert, Julian C. Carter, Selina Begum, Ruth De Vos, Paul Kalra, Matthew Quint, Kayode Adeniji, Thomas P. Brown, Ameera X. Patel, Anoop J. Chauhan

**Affiliations:** a Respiratory Department, Portsmouth Hospitals University NHS Trust, Portsmouth, UK; b Machine Learning / Hardware / Clinical Departments, TidalSense Limited, Cambridge, UK

**Keywords:** Capnography, breathlessness, asthma, chronic heart failure, pneumonia

## Abstract

**Background:**

In an increasingly global comorbid population, there are significant challenges to diagnosing the cause of breathlessness. Furthermore, once chronic respiratory conditions have been diagnosed, there is considerable difficulty in detecting deterioration early enough to provide timely, effective intervention. Current methods of diagnosing and monitoring conditions that cause breathlessness, such as asthma and chronic heart failure, can be extensive and difficult to perform.

**Methods:**

This observational, proof-of-concept study explored the potential of high-resolution capnography to differentiate respiratory and cardiac conditions causing breathlessness. Using an early model of the N-Tidal device, we analysed capnography waveforms from participants with severe asthma, chronic heart failure, and pneumonia, and compared them to healthy baseline controls.

**Results:**

A moderate correlation was observed between alpha angle and spirometry metrics in asthma (r^2^ = 0.48 for FEV_1_/FVC), along with a moderate association between alpha angle and left ventricular ejection fraction in heart failure (r^2^ = 0.46). Pneumonia recovery was marked by a 12.6% median increase in end-tidal CO_2_.

**Conclusion:**

These results suggest that high-resolution capnography may offer promise for non-invasive diagnosis and monitoring of cardiorespiratory conditions.

**Trial registration:**

NCT03356288 (registered on 7 September 2017)

## Introduction

Respiratory and cardiac diseases causing breathlessness are both highly prevalent and major causes of healthcare utilisation across the UK. Approximately 8 million people in the UK, or 12% of the population, are affected by asthma alone [1]. Chronic heart failure (CHF) affects over half a million individuals, while community-acquired pneumonia (CAP) affects up to 1% of adults annually [2,3]. Patients with acute or chronic cardiorespiratory conditions may experience symptom deterioration, and even with a clear diagnosis, often struggle to determine when to seek medical advice. Furthermore, many patients have multiple factors contributing to their breathlessness, with conditions such as dysfunctional breathing being an under-recognised component. Consequently, differentiating the true driver of a patient’s breathlessness is increasingly challenging. The combined burden of these conditions on the healthcare economy is substantial, directly accounting for over £2 billion per annum, with an indirect societal cost in excess of £6 billion in the United Kingdom [4–6].

Current methods of diagnosing and monitoring breathlessness, such as spirometry, peak flow, transthoracic echocardiography (TTE), imaging and arterial blood gas (ABG) tests, can be invasive or difficult to perform. Furthermore, these tests can often be difficult to access in primary care and most cannot be used by patients at home, making it challenging for patients to self-monitor and engage with the management of their disease. Consequently, there is often poor compliance associated with the usage of self-management techniques [7]. There is a need for simple, effort-independent tools that can support healthcare professionals in diagnosing conditions that cause breathlessness and help patients in monitoring their own health. Ideally, these tools should empower patients to take ownership of their condition, maintain control, and ultimately enhance their overall health and well-being.

Capnography, predominantly used in anaesthetics, critical care and emergency departments, enables monitoring of respired carbon dioxide concentration, providing near-real-time information about ventilatory status and airway patency across all stages of the respiratory cycle. The N-Tidal device, developed by TidalSense, is a lightweight, handheld capnometer that captures data in 75 seconds of tidal breathing, enabling carbon dioxide (CO_2_) measurement with greater sampling frequency, reliability and accuracy than previously possible [8]. This study aims to explore the characteristics of capnography waveforms measured by an early model of the N-Tidal device (the NTC1 device) to determine their potential in differentiating between different respiratory and cardiac conditions and healthy controls.

## Methods

### Study design and datasets

The General Breathing Record Study (GBRS) was a six-month longitudinal observational study conducted at Queen Alexandra Hospital (QAH) in Portsmouth from 9 August 2017 to 4 July 2018. The study recruited participants with clinically confirmed diagnoses of severe asthma, CHF, breathing pattern disorder (BPD), pneumonia, motor neurone disease, as well as healthy controls with no history of lung or cardiac disease. Exclusion criteria included any other lung conditions, chest wall issues, neuromuscular problems, heart conditions, or any other comorbidities that could impact spirometry, lung function tests, or capnography measurements. Participants deemed likely to have difficulty consistently completing study procedures over the six-month period were also excluded. The complete eligibility criteria for each condition are detailed in the published study protocol [9].

All participants provided informed consent prior to enrolment. Data handling adhered to applicable data protection legislations, including the EU/UK General Data Protection Regulation. The South Central – Berkshire Research Ethics Committee (REC) granted ethical approval for the study, which was registered on ClinicalTrials.gov (NCT03356288).

### Study procedures

Capnography data was collected using an early model of the N-Tidal device (called the NTC1 device). Participants were instructed to use the device twice daily (morning and evening), with the option to increase usage to 6 times daily if they experienced worsening breathlessness or felt unwell. The NTC1 device is designed to accurately and reliably record respired partial pressure of CO_2_ (pCO_2_) directly from the mouth. Eligible participants received a NTC1 device for home use after undergoing comprehensive training on its correct operation and handling. During each measurement, participants were instructed to perform normal tidal breathing through the device’s mouthpiece for 75 seconds. This duration was chosen to ensure capture of a sufficient number of breath cycles for analysis. To ensure data quality and participant compliance, the research team maintained regular contact with all study participants throughout the six-month study period. This ongoing communication allowed for prompt addressing of any issues or concerns, and reinforcement of proper device usage techniques. The study design incorporated a longitudinal approach, enabling the collection of data over time to capture potential variations in capnography waveforms associated with disease progression and treatment effects.

Additional disease-specific data, e.g. spirometry for asthma participants, C-reactive protein (CRP) for pneumonia participants, were collected at baseline, 2, 4 and 6 months, and at point of exacerbation, as detailed in the study protocol [9].

### Feature engineering

Each capnogram underwent a comprehensive pre-defined pre-processing pipeline to ensure rigorous data quality control for analysis. Initially, denoising techniques were applied to remove high-frequency artefacts, followed by breath separation to distinguish individual breaths within the capnogram. The breath phase segmentation process then isolated specific phases of each breath cycle, allowing for detailed geometric analysis of waveform characteristics. Anomalous breaths were identified and excluded automatically using predefined signal-quality criteria embedded within the processing pipeline. These algorithms were developed prior to and independently of the present analyses and were applied uniformly across all recordings. This process addressed artefacts related to environmental factors (e.g. condensation), involuntary participant actions (e.g. coughing or swallowing), and cardiogenic oscillations, ensuring objective and reproducible data quality control.

**Figure 1.**
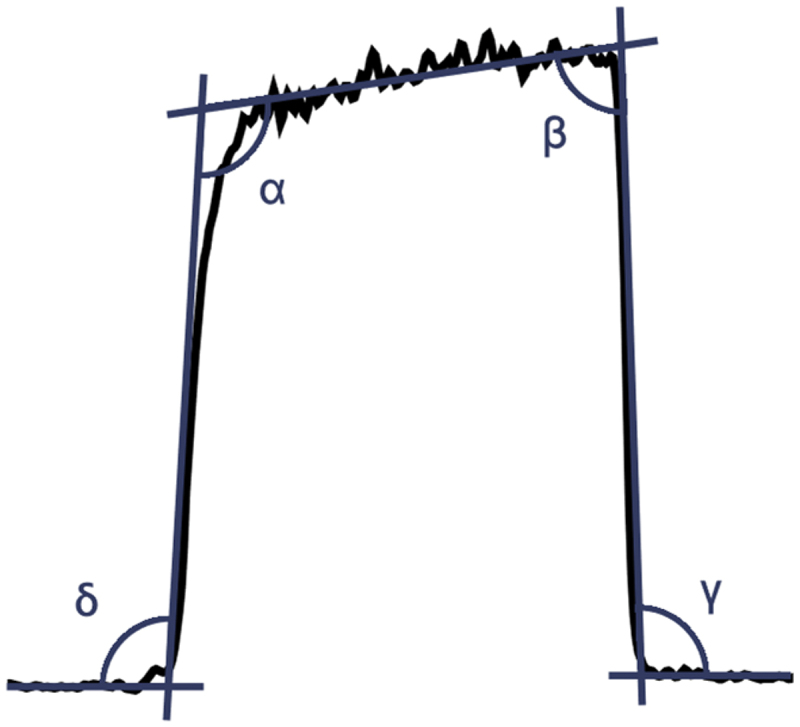
Illustration of a typical breath waveform and its salient angles and gradients from their corresponding phases.

Feature extraction was performed on the processed capnograms, yielding two main categories of features: per-breath features and whole-capnogram features. Per-breath features captured geometric characteristics such as angles (e.g. α, β, γ, δ) and gradients associated with specific waveform phases, while whole-capnogram features included metrics such as respiratory rate [10–13]. Figure 1 illustrates a typical breath waveform captured by the NTC1 capnometer, with its associated phases, gradients, and angles. A total of 87 per-breath features and 5 whole-capnogram features were extracted. To account for variability in respiratory rates, an additional set of features was derived by normalising breaths in the time domain using linear interpolation. This ensured that geometric differences in waveforms were not confounded by differences in breathing patterns. For each capnogram, per-breath features were aggregated using both median and standard deviation values, resulting in a final dataset comprising 159 distinct features per capnogram. These features provided a robust foundation for subsequent statistical analyses aimed at distinguishing between disease cohorts and healthy controls.

### Statistical analysis

Statistical analysis was performed using Python (version 3.12.2) as well as open-source scientific libraries such as SciPy (1.2.2), NumPy (1.25.0) and Pandas (1.5.3). Summary statistics were presented as medians with interquartile ranges (IQR) for continuous variables, and frequencies and percentages for categorical variables. Differences in distributions for continuous variables were assessed using the Mann-Whitney U test, with adjustments for multiple comparisons using the Bonferroni correction. Effect size testing was performed using Cohen’s *d* test to quantify the magnitude of differences between groups. To evaluate the strength of relationships between continuous variables, Pearson’s correlation coefficient (*r*) was calculated. This metric evaluated linear associations between capnography features and clinical parameters, such as spirometry measurements in asthma participants and cardiac function indicators in heart failure participants. These approaches ensured a comprehensive assessment of the discriminatory power of various capnography features across different respiratory and cardiac conditions.

## Results

### Demographics and baseline characteristics

A total of 10,085 capnograms were collected from 58 participants with the following diagnoses: asthma, CHF, pneumonia, and healthy controls. Of those, five withdrew from participation. Following dataset quality control, applied through the pre-defined pre-processing pipeline, 3,640 capnograms from 50 participants were used for data analysis. A large number of capnograms were automatically excluded due to the presence of condensation artefacts which the older model of the N-Tidal device (the NTC1 device) used in this study was prone to; this issue has been resolved in newer hardware. Of remaining capnograms, the average breath quality was 96.0% for asthma participants, 98.0% for CHF participants, 97.4% for healthy participants and 96.7% for pneumonia participants. The baseline characteristics of these participants are presented in Table 1, which provides a comprehensive overview of the study population. This table includes key demographic information such as age, sex distribution, and relevant clinical parameters specific to each disease cohort. There was no systematic bias identified in excluded datasets, and the quality-controlled dataset contained sufficient representation across diseases, and demographics. Adherence to twice-daily data collection as per the protocol was 75.9% in the asthma cohort, 78.1% in the CHF cohort, 73.3% in the healthy cohort, and 26.0% in the pneumonia cohort.

**Table 1.** Comparison of the baseline characteristics of each disease cohort in the study population. Categorical data is represented as a number with its percentage of the total (n (%)). Continuous data is represented as (median (IQR)).

	Healthy (*n* = 10)	Asthma (*n* = 17)	CHF (*n* = 9)	Pneumonia (*n* = 14)
**Age**	36.0 (29.3–44.3)	57.0 (49.0–61.0)	72.0 (66.0–75.0)	56.5 (43.3–66.8)
**Sex (female)**	6 (60.0%)	10 (58.8%)	2 (22.2%)	9 (64.3%)
**BMI**	25.9 (22.1–31.9)	30.5 (25.5–35.0)	27.0 (24.1–30.3)	25.8 (24.2–31.2)
**Smoking History**				
Current smoker	0 (0.0%)	2 (11.8%)	2 (20.0%)	4 (28.6%)
Ex-smoker	3 (30.0%)	6 (35.3%)	8 (80.0%)	8 (57.1%)
Never smoked	7 (70.0%)	9 (52.9%)	0 (0.0%)	2 (14.3%)
**Pack Years**	0.0 (0.0–0.5)	0.0 (0.0–5.0)	30.0 (11.0–45.0)	10.0 (3.7–15.0)
**Spirometry**				
FEV_1_/FVC (%)	81.0 (78.8–84.4)	70.5 (59.0–74.5)	–	–
Pred. FEV_1_ (%)	107.8 (98.8–113.1)	82.0 (57.5–101.5)	–	–

### Characterisation of asthma-related airway obstruction in the CO_2_ waveform

First, a systematic comparison was performed between the CO_2_ features in the asthma cohort and the healthy cohort with a view to understanding the parts of the CO_2_ waveform that are most discriminative of asthma. As shown in Table 2, the strongest effect size (measured by Cohen’s *d*) was seen in features from the alpha angle region, consistent with previous research that has highlighted the importance of this region in quantifying the severity of airways obstruction [14,15]; the most discriminating two alpha angle features had large effect sizes with Cohen’s *d* = 1.16 and 1.14 respectively.

**Table 2.** Counts and proportions of region-specific features in each effect size category (determined by Cohen’s *d*) between the asthmatic and healthy cohorts.

Region	Features by effect size (as measured by Cohen’s *d*)		
	Small *d* = 0- 0.49	Medium *d* = 0.5- 0.79	Large *d* ≥ 0.8
Alpha	5 (41.7%)	5 (41.7%)	2 (16.7%)
Beta	8 (72.7%)	3 (27.3%)	0
Gamma	6 (66.7%)	3 (33.3%)	0
Delta	9 (100%)	0	0

**Figure 2.**
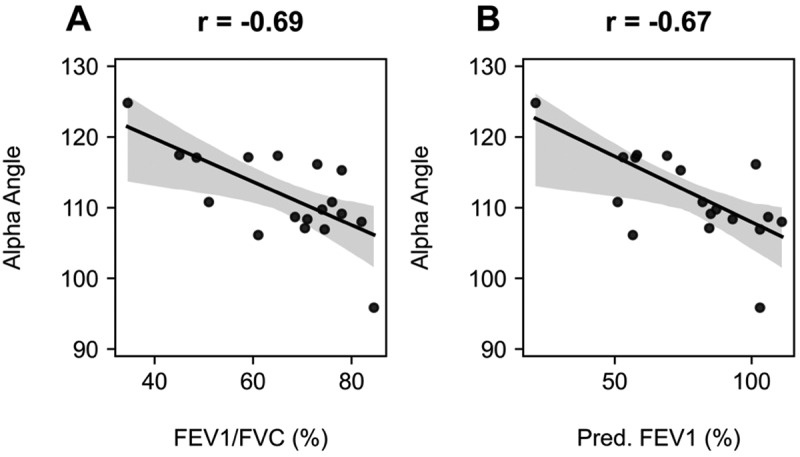
Regression plots of the alpha angle against (A) FEV_1_/FVC and (B) % predicted FEV_1_ for the asthma cohort. The alpha angle demonstrated strong correlations against both spirometry metrics (*r* of (a) −0.69 and (b) −0.67).

Next, the relationship between alpha angle features and spirometry metrics was investigated to determine whether there was evidence of physiologic grounding of alpha angle variability and airflow limitation as measured by FEV_1_/FVC ratio and % predicted FEV_1_. Baseline spirometry data from 18 asthmatic participants was analysed in relation to the alpha angle. Results revealed moderate negative correlations between the alpha angle and both FEV_1_/FVC ratio (*r* = −0.69, *p* = 0.0014) and % predicted FEV_1_ (*r* = −0.67, *p* = 0.0025), as illustrated in Figure 2. These findings may suggest that a larger alpha angle is associated with a greater degree of airway obstruction, consistent with hypothesis.

### Relationship of blood biomarkers and cardiac echocardiography against CO_2_-related metrics in CHF

**Figure 3.**
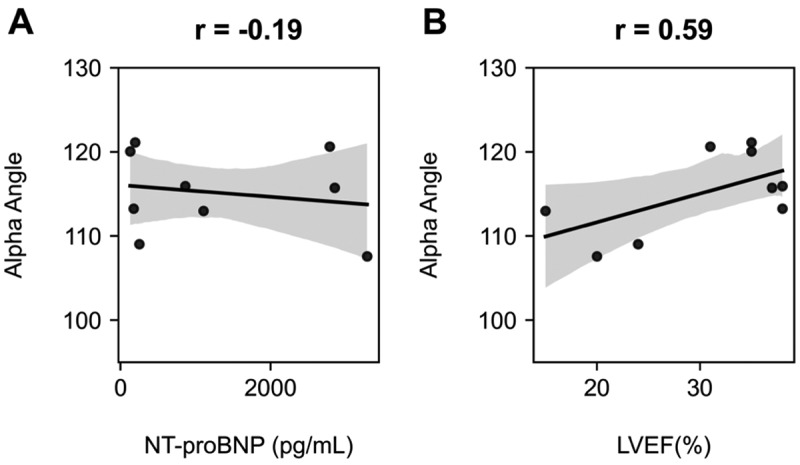
Regression plots of the alpha angle against (A) NT-proBNP and (B) LVEF for the CHF cohort. The alpha angle demonstrated a poor correlation against NT-proBNP but a good correlation with LVEF.

Next, we examined the relationships between capnography features and key cardiac biomarkers in CHF participants used to diagnose and monitor disease progression. The analysis focused on two primary metrics: N-terminal pro b-type natriuretic peptide (NT-proBNP) and left ventricular ejection fraction (LVEF). No significant correlations were found between any capnography features and NT-proBNP levels. However, a notable finding emerged when examining the relationship between capnography and cardiac function. A positive association was observed between the alpha angle of the capnogram and LVEF, with a correlation coefficient (r^2^) of 0.46. This correlation indicates that the alpha angle, which represents the transition of gas from larger to smaller airways, may provide valuable insights into cardiac function in CHF participants. Figure 3 illustrates these relationships, showing the regression plots of the alpha angle against NT-proBNP and LVEF. The contrasting results between NT-proBNP and LVEF correlations highlight the complex interplay between respiratory mechanics and cardiac function in CHF. While these findings are exploratory, they suggest that capnography may offer complementary physiological information alongside traditional biomarkers.

### Tracking recovery from pneumonia using capnography features

Here we investigated the relationship between capnography features and recovery from pneumonia. By the end of the study, 12 out of 17 pneumonia participants (70.5%) showed CXR-confirmed resolution of their pneumonia. The average breath waveform of pneumonia participants during the first week after diagnosis is illustrated in Figure 6D. This waveform exhibited a lower average alpha angle, more closely resembling the healthy waveform. The key point of interest in the capnogram was the end-tidal CO_2_, expected to be low at the onset of pneumonia, consistent with type I respiratory failure.

**Figure 4.**
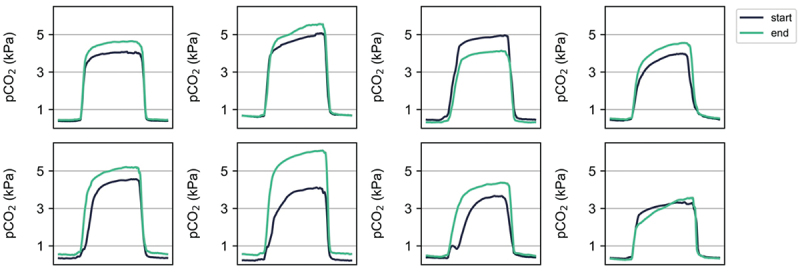
Average standardised breath waveforms from the first week after a confirmed diagnosis of pneumonia (dark blue), and a week prior to the end of the study after pneumonia was cleared (green), displayed for the eight participants for which the data was available. The breath waveforms illustrate the increase in end-tidal CO_2_ upon the resolution of pneumonia, which was the case in all subjects but one.

**Figure 5.**
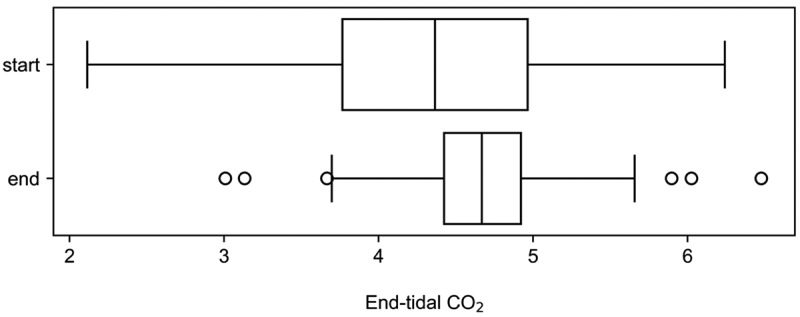
Boxplot of end-tidal CO_2_ for the first week after a confirmed diagnosis of pneumonia (start), and a week prior to the end of the study after pneumonia was cleared (end). The median increase in the end-tidal CO_2_ after the clearance of pneumonia was 12.6% (*p* = 0.01).

To visualise changes in capnography features accompanying recovery, two average standardised waveforms were examined for each participant: one from the first week after confirmed pneumonia diagnosis, and another from the week prior to study end when the participant was cleared of pneumonia. Due to poor adherence during convalescence in this particular subgroup and data quality, only eight participants recorded data in their final week on the study. Figure 4 displays these waveforms, revealing an increase in average end-tidal CO_2_ upon pneumonia resolution in all but one subject. Figure 5 illustrates the distributions of end-tidal CO_2_ for these defined time intervals. The median increase in end-tidal CO_2_ after pneumonia clearance was 12.6% (*p* = 0.01), indicating that the change in end-tidal CO_2_ level may provide a useful marker for tracking pneumonia recovery using capnography, offering a non-invasive method to monitor participant progress over time.

### CO_2_ waveform shape varies in cardiorespiratory disease states

**Figure 6.**
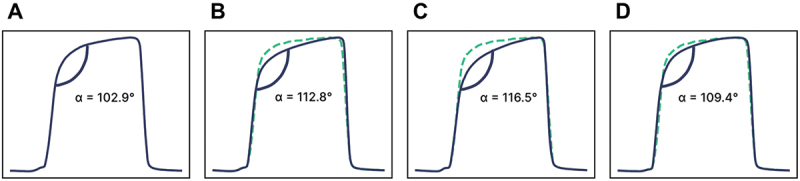
Average standardised breath waveforms of the (A) healthy, (B) severe asthma, (C) CHF, and (D) pneumonia cohorts. The median alpha angle for each cohort is 102.9°, 112.8°, 116.5°, and 109.4° respectively. Each waveform of a disease cohort has a healthy waveform overlayed in green.

The study revealed distinct variations in CO_2_ waveform shapes across different cardiorespiratory disease states. The typical waveform of a healthy subject is characterised by sharp transitionary upstrokes and downstrokes. In contrast, participants with asthma and congestive heart failure – conditions associated with obstructive respiratory physiology – demonstrated notable variations in waveform geometry, particularly a steeper expiratory plateau phase, as shown in Figure 6.

**Figure 7.**
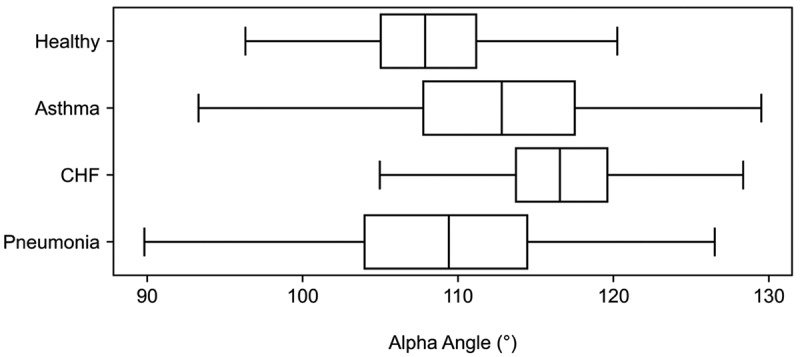
Boxplots of the alpha angle for the healthy controls, asthma, CHF, and pneumonia subjects. Statistically significant differences (*p* < 0.01) in the alpha angle were observed between both the asthma and CHF participants compared to the healthy controls.

Statistical analysis using hypothesis testing between each of the asthma and CHF cohorts against the healthy group indicated significant differences in the alpha angle (*p* < 0.01 for each comparison). Post-hoc calculation of Cohen’s *d* revealed a medium effect size for the comparison between healthy vs. CHF (*d* = 0.55) and a very high effect size for the comparison between healthy vs. asthma (*d* = 1.74). These findings also support the potential of the alpha angle as a discriminatory feature in distinguishing between healthy individuals and those with certain respiratory conditions. Figure 7 provides a visual representation of the alpha angle distributions across different cohorts including healthy controls, asthma, CHF and pneumonia participants.

The median alpha angles for each cohort were 102.9°, 112.8°, 116.5°, and 109.4° for healthy, severe asthma, CHF, and pneumonia, respectively. These distinct patterns in waveform characteristics suggest that capnography could potentially serve as a valuable tool in differentiating between cardiorespiratory conditions, based on the underlying physiologic changes associated with each disease state.

## Discussion

This study explored the potential of high-resolution capnography using an early model of the N-Tidal capnometer (the NTC1 device) to differentiate between various respiratory and cardiac conditions causing breathlessness. By establishing a healthy capnogram profile as a reference, we were able to identify distinct waveform characteristics associated with severe asthma, CHF, and pneumonia. The healthy capnogram profile, characterised by a smooth, symmetric waveform with sharp transitions between inspiratory and expiratory phases, provided a clear benchmark for comparison. This ‘square-like’ waveform geometry reflects unobstructed airways and healthy lung morphology, serving as a valuable baseline for assessing pathological changes and comparisons between different cardio-respiratory conditions.

In severe asthma, we observed a larger alpha angle and prolonged phase 2 duration, indicative of airways obstruction. The strong negative linear correlation between the median alpha angle and both FEV_1_/FVC ratio and predicted FEV_1_ suggests that the N-Tidal capnometer can quantify airway obstruction severity. This finding has implications for asthma management, potentially offering a non-invasive, effort-independent alternative to traditional spirometry.

CHF participants exhibited an even greater alpha angle curvature compared to the asthma cohort. We hypothesise that this may be due to pulmonary oedema affecting carbon dioxide clearance through ventilation-perfusion mismatch [16]. While the alpha angle demonstrated a positive correlation with LVEF, it showed no association with NT-proBNP, suggesting that capnography may not directly reflect the neurohormonal changes indicated by NT-proBNP in CHF participants. This discrepancy warrants further investigation and highlights the complex interplay between biomarker cardiac function and respiratory mechanics in heart failure.

The pneumonia cohort presented a unique pattern, with a slight curvature of the alpha region on initial presentation, less pronounced than in asthma or CHF. This characteristic aligns with the nature of pneumonia which typically does not cause airflow obstruction. The subtle curvature likely reflects the acute accumulation of inflammatory exudate in the alveoli, impairing gas exchange. The observed increase in end-tidal CO_2_ levels during recovery is consistent with the resolution of type 1 respiratory failure and normalisation of breathing patterns. Although clinical recovery from pneumonia in otherwise healthy individuals is generally uncomplicated, resolution of symptoms does not necessarily equate to full physiological recovery. The increase in end-tidal CO_2_ observed during convalescence may reflect temporary changes in ventilation-perfusion dynamics that persist beyond clinical improvement. While these findings require further validation, they suggest that end-tidal CO_2_ may capture subtle physiological changes during recovery acute respiratory infection.

While our findings are promising, there are several limitations. The small sample size in each disease cohort restricts the generalisability of our results. The analyses should therefore be considered exploratory and hypothesis-generating rather than confirmatory. The study was not designed to provide precise effect size estimates or to establish clinically actionable thresholds. Statistical comparisons were included to identify preliminary group-level differences and associations that may inform the design of future, adequately powered studies. Accordingly, reported *p* values should be interpreted cautiously and in the context of the descriptive data. Future studies should aim for larger, more diverse patient populations to validate these findings. The quantity of missing or poor-quality data presented challenges in longitudinal analysis, potentially introducing bias. This is largely due to the presence of artefacts in the older version of the N-Tidal device (the NTC1 device) used in this study; this issue has been resolved in newer models. There were also challenges with participant adherence in the pneumonia cohort during the convalescence phase, which limited the interpretability of these findings. We hypothesise that this was likely due to reduced motivation to collect data once participants began to feel clinically better.

The longitudinal nature of our study, while providing valuable insights into disease progression and recovery, also introduced challenges in maintaining consistent data collection over the six-month period. Factors such as participant fatigue, changes in disease state, or concurrent treatments may have influenced measurement consistency. Future studies should consider incorporating more frequent clinical assessments to correlate capnography changes with other physiological parameters and treatment responses.

In summary, high-resolution capnography shows promise as a tool for measuring pulmonary function and disease. By enabling effort-independent measurements, it has the potential to enable data capture in a wider variety of patients who may not be able to perform traditional pulmonary function testing. However, further studies are necessary to confirm its clinical utility in longitudinal monitoring of cardiorespiratory disease. As healthcare moves towards more personalised and home- and community-based care models, technologies such as the N-Tidal capnometer could play an important role in improving the management of chronic respiratory and cardiac conditions, ultimately enhancing patient outcomes and quality of life.

## Data Availability

The datasets generated during and/or analysed during the current study, and any algorithms developed, are not publicly available for data protection, confidentiality, and commercial sensitivity reasons.
